# “Groupitizing”: A Visuo-Spatial and Arithmetic Phenomenon

**DOI:** 10.1162/opmi_a_00181

**Published:** 2025-01-20

**Authors:** Paula A. Maldonado Moscoso, Giovanni Anobile, Giuseppe Maduli, Roberto Arrighi, Elisa Castaldi

**Affiliations:** Center for Mind/Brain Sciences, University of Trento, Trento, Italy; Department of Neuroscience, Psychology, Pharmacology, and Child Health, University of Florence, Florence, Italy

**Keywords:** groupitizing, ANS, subitizing, cross-modal attention

## Abstract

When objects are grouped in space, humans can estimate numerosity more precisely than when they are randomly scattered. This phenomenon, called groupitizing, is thought to arise from the interplay of two components: the subitizing system which identifies both the number of subgroups and of items within each group, and the possibility to perform basic arithmetic operations on the subitized groups. Here we directly investigate the relative role of these two components in groupitizing via an interference (dual task) paradigm. Participants were required to estimate numerosities of grouped and ungrouped arrays while their attentional resources were fully available (single task) or while performing concurrent tasks loading auditory or visuo-spatial attention (both known to interfere with the subitizing process) as well as while performing arithmetic calculation. The attentional cost of performing any concurrent task was overall higher for grouped than ungrouped stimuli, supporting the idea that groupitizing relies on the recruitment of more than one attention-dependent mechanism. However, depriving visuo-spatial attention and preventing participants from performing calculations caused the strongest decrement in sensory precision for grouped numerosities indicating that these attentional components play a major role in groupitizing. These results are in line with the existence of an estimation mechanism that might operate across all numerical ranges, supplemented by attentional mechanisms (subitizing). This study shows that this attentional-demanding mechanism can be activated also when processing numerosities outside of the subitizing regime (*n* > 4), provided that grouping cues are available and, in concert with calculation abilities, gives rise to the groupitizing phenomenon.

## INTRODUCTION

Humans, as well as non-human primates, can rapidly estimate the number of objects in a scene without counting. Two systems are thought to support this ability: the subitizing system and the Approximate Number System (ANS; Dehaene, 2011). The former guarantees extremely fast and errorless numerical judgments for quantities up to four elements (Jevons, 1871; Kaufman et al., 1949), whereas larger numerosities are estimated slowly and approximately through the ANS (Dehaene, 2011). While there is no consensus on whether or not the subitizing and ANS are neatly separated processes, several studies reported that they are at least highly intertwined. The crosstalk between these two systems can be particularly appreciated whenever an experimental manipulation alters the typical numerical range over which each system is thought to operate. For example, under dual-task or attentional-blink paradigms, the precision of numerosity estimation in the subitizing range decreases to the level of the one typically observed for larger numerosities processed by the ANS (Burr et al., 2010; Vetter et al., 2008). Vice versa, a recently described phenomenon called “Groupitizing”, was found to *extend* the subitizing range by improving the coding efficiency of arrays with more than 4 items (Maldonado Moscoso et al., 2020). This phenomenon occurs whenever the items to be enumerated are arranged into groups rather than randomly scattered in space. Groupitizing was first observed by Starkey and McCandliss (2014) who showed that numerical estimates of quantities exceeding the subitizing range were more rapid if items were grouped into small clusters each containing a number of items comprised within the subitizing range. Recent studies found that, other than response time, groupitizing also increases sensory precision (Anobile et al., 2020; Ciccione & Dehaene, 2020). The groupitizing advantage can be observed for several grouping cues irrespective of stimulus format (simultaneous and sequential; Anobile et al., 2020) and sensory modality (visual and auditory; Anobile et al., 2021). Groupitizing is still a relatively unexplored phenomenon, yet the results from the few existing studies have led researchers to hypothesize that it might arise from the interplay of at least two processes: the activation of the subitizing system and the application of arithmetical operations on the subitized groups.

In line with the involvement of the subitizing system during groupitizing, a study found that presenting clustered arrays for only 150 ms was sufficient for adult participants to extract both the number of groups and the number of dots within each group, pointing at the recruitment of processes able to track multiple objects in parallel (Wege et al., 2022). Furthermore, Caponi et al. (2023) recently showed that a very early and negative component (N1) of the event-related potentials was modulated in a similar fashion by both the number of subgroups in clustered arrays and by numerosities in the subitizing range, suggesting that clustered elements might activate the subitizing system at a very early stage.

In addition to the recruitment of the subitizing system, another important component thought to subtend the groupitizing phenomenon is the automatic application of arithmetical strategies: the subitized items might be entered as operands in mental sums or multiplications to precisely enumerate the total quantity of the whole array (Ciccione & Dehaene, 2020). A similar hypothesis was first advanced by Starkey & McCandliss who observed that the groupitizing advantage did not emerge before formal schooling has started (Starkey & McCandliss, 2014) and that it increased during schooling until early adolescence (Guillaume et al., 2023; Starkey & McCandliss, 2014), thereby suggesting that mastering basic arithmetic principles might favour the emergence of the groupitizing advantage. Further evidence in support to the relationship between groupitizing and arithmetic comes from a recent study showing that the groupitizing advantage was stronger in university students with excellent performance in basic mathematics compared to those with lower math proficiency (Ciccione & Dehaene, 2020). Finally, a recent fMRI study found that enumerating grouped quantities specifically elicited the activation of brain regions (bilateral fronto-parietal network and bilateral angular gyrus) involved in calculation/arithmetical fact retrieval compared to enumerating random arrays (Maldonado Moscoso, Greenlee, et al., 2022).

Overall, the evidence collected so far, suggests that the groupitizing phenomenon relies on both the recruitment of the subitizing system and calculation abilities (Anobile et al., 2020; Ciccione & Dehaene, 2020; Guillaume et al., 2023; Maldonado Moscoso et al., 2020; Starkey & McCandliss, 2014).

In the current study we tested the presumed role of these two components, subitizing and calculation abilities, by determining whether and to what extent groupitizing advantage is attenuated when direct interference prevents leveraging these components.

We moved from previous evidence showing that the precision of the subitizing system is more strongly affected by attentionally demanding concurrent tasks compared to ANS (Anobile et al., 2012; Burr et al., 2010). Indeed it was shown that, although depriving visual attention had the strongest impact (Pomè, Anobile, Cicchini, Scabia, et al., 2019), loading auditory or tactile attention also hampered subitizing to indicate it has to be considered an a-modal system (Anobile et al., 2012). We reasoned that if subitizing plays a key role in groupitizing, then the latter should be susceptible to manipulation of multisensory attention too. To test this hypothesis, we asked participants to enumerate grouped or ungrouped arrays while performing concurrent dual tasks loading visual or auditory attention. We expect the concurrent tasks to affect sensory precision of grouped arrays more than that of ungrouped arrays, mirroring the stronger impact they had on subitizing than on the ANS (Pomè, Anobile, Cicchini, Scabia, et al., 2019). More, we also expect interference to occur irrespective of the sensory modality the deprived attentional resources belong to (auditory or visual, in line with Anobile et al., 2012) despite previous results might also support an unequal weight of auditory and visuospatial attentional resources, with the latter playing a more critical role (in line with Pomè, Anobile, Cicchini, Scabia, et al., 2019).

Next, we tested whether having the possibility to perform arithmetic operations on the subitized clusters is necessary to yield the groupitizing advantage on the total ensemble. To this aim we interfered with calculation by asking participants to estimate numerosities of random or grouped arrays while solving arithmetical operations (not related to the possible operations they might be carrying out to combine the clusters). If participants automatically perform basic arithmetic operations during numerosity estimation of grouped stimuli, by asking them to solve arithmetic calculations we should interfere with the basic mathematical processes and thus, be able to hamper or annul the groupitizing advantage.

## MATERIALS AND METHODS

### Power Analysis

Sample size was calculated with a-priori Power analysis using G*Power software (version 3.1). The main goal of the current experiment was to detect a change on the numerosity sensory precision under attentional load. For this reason, the analysis aimed to calculate the required sample size to reliably detect a significant difference between the numerosity sensory precision of grouped stimuli in the single task and in the dual task in a two tailed paired *t*-test. The effect size was estimated from Maldonado Moscoso et al. (2020) with a *α* = 0.05 / 28 (number of comparisons of grouped and ungrouped conditions among the 4 tasks conditions) and a Power of 0.95, the analysis suggested a required sample size of 28.

### Participants

Twenty-eight young adults (mean age = 25.1, standard deviation = 3.4, range = 19–31; 2 authors) with normal or corrected-to-normal vision participated in the study. Participants were all university students with no mathematical or other learning disorders nor over-exercised calculation skills. The research was approved by the ethics committee (Commissione per l’Etica della Ricerca, University of Florence, July 7, 2020, n. 111). The research was performed in accordance with Declaration of Helsinki and informed consent was obtained from all participants prior the experiment.

### Materials and Procedure

**Figure 1.**
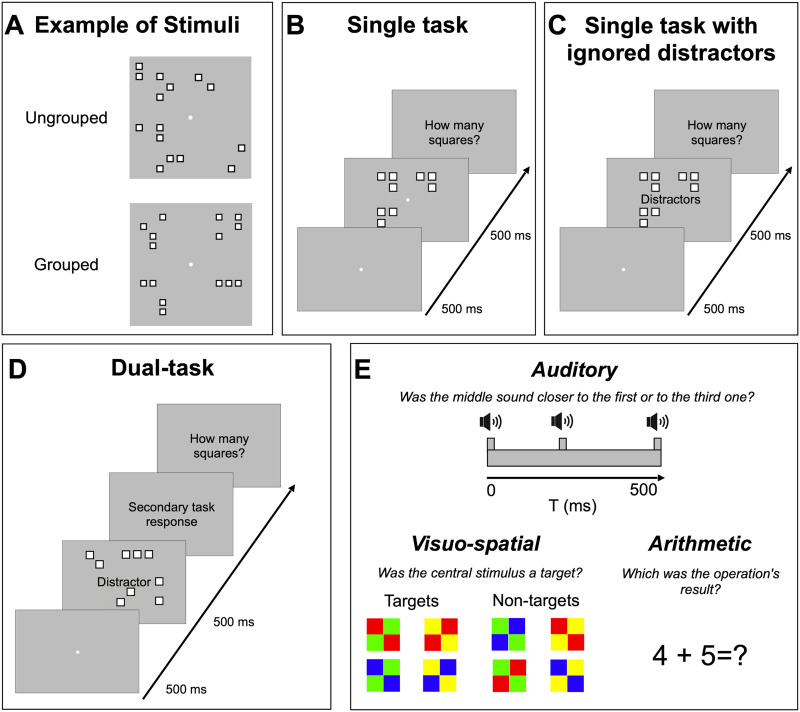
Illustration of the stimuli and procedure. (**A**) Example of ungrouped and grouped stimuli. (**B**, **C**, **D**) Each trial started with a central fixation point, followed by a briefly flashed ensemble of squared items (500 ms). (**B**) Single task: participants were asked to verbally report the perceived numerosity of arrays of squares. (**C**) In the single task with distractors condition, we presented grouped stimuli at the same time with three distractors: auditory, visuo-spatial and arithmetic stimuli. Participants verbally reported the perceived numerosity and ignored the distractors. (**D**) Dual task: participants first responded to the concurrent task, then verbally reported the perceived numerosity. (**E**) For the concurrent tasks we used auditory and visual stimuli. Auditory attention was loaded by means of an auditory bisection task, while visual attention was loaded by means of color-conjunction task. Finally, we interfered with calculation by asking participants to solve simple operations. Auditory stimuli were provided through headphones, visual stimuli were displayed in the center of the screen.

Participants sat 57 cm from a 59″ screen monitor (60 Hz), in a quiet and dimly lit room. They performed a numerosity estimation task in both single and dual task conditions. For each condition (single and dual task) participants were asked to estimate the numerosity of ungrouped and grouped arrays of squares (ranging from 5 to 16; Figure 1A). The conditions were tested separately with the order counterbalanced across participants. No feedback was provided, and participants were not informed about the numerical range. They were also not informed about the different spatial structures of the numerical arrays (ungrouped or grouped).

As in the previous studies (Anobile et al., 2020; Maldonado Moscoso et al., 2020), we asked participants to estimate numerosity of ungrouped and grouped stimuli in single task (Figure 1B). To disentangle the role of mere sensory load to attentional resources deprivation, grouped stimuli were also presented together with auditory, visuo-spatial or arithmetic distractors (Figure 1C), the same we used in the dual tasks (single task with distractors condition; see details of distractors below). In the single task with distractors condition participants were asked to ignore the distractors and verbally estimate the numerosity of the arrays showed onscreen. The answers were recorded by the experimenter who hit the spacebar when the participant responded and then entered the answer on the numeric keypad, which initiated the next trial. Response time was measured from the stimulus offset to the beginning of vocalization (indicated by experimenter’s keypress). Participants were asked to respond as soon as possible, but also to concentrate on accuracy.

In the dual task conditions (Figure 1D), grouped and ungrouped stimuli were again tested separately. Participants performed three separate sessions in which they were asked to perform the numerosity estimation task together with a concurrent task based on auditory, visuo-spatial and arithmetic distractors (Figure 1E).

Each session consisted of 8 blocks (4 with grouped and 4 with ungrouped stimuli) of 36 trials each, except for the single task with distractors session for which we tested only the grouped condition (4 blocks of 36 trials each). Participants completed on average 432 trials for the single task and 864 for the dual task sessions, for a total of 1296 trials for the entire experiment. Trials with response times and responses higher and lower than 3 standard deviations were considered outliers and eliminated from the analysis (0.6%).

### Stimuli and Experimental Paradigm

Stimuli were generated and presented with PsychToolbox routines for Matlab (ver. 2016b, The Mathworks, Inc.). Stimuli were arrays of squares (0.4° × 0.4°) displayed for 500 ms on each trial. The items were white squares with black borders (so that luminance was not a cue to number). Squares could not overlap and were constrained to fall within a 6° × 6° virtual square area (Figure 1A).

In the ungrouped condition, the position of each square was randomly selected from 106 possible positions (within the stimulus area). These positions corresponded to the centers of equally spaced sectors in a 6° × 6° area aligned with the fixation point, with each grid cell measuring 0.54° × 0.54°. To prevent squares from being too close to the fixation point, we maintained a 2.73° × 1.64° area around the fixation point where no squares were presented. For the grouped condition, stimuli were arranged in 4 possible quadrants, each measuring 1.64° × 2.2°, and centered at ∼3° from central fixation point. Each square could appear in one of the 12 possible locations within each quadrant. Each group was first randomly assigned to one quadrant (between 1 and 4), then the individual items positions were randomly selected between one of the 12 in the selected quadrant.

Participants estimated numerosities between 5 to 16. In the ungroup condition, the squares were randomly located in one of the possible locations on every trial. In the grouped condition, each numerosity was organized into 2 to 4 subgroups, each containing a variable number of squares (between 1 and 5). The configurations used for each numerosity were as follows: N5: 2,2,1; 3,2; 1,1,1,2; N6: 3,3; 2,2,2; 3,2,1; N7: 3,3,1; 2,2,2,1; 3,2,1,1; N8: 3,3,2; 2,2,2,2; 4,4; N9: 4,3,2; 4,4,1; 3,3,3; N10: 3,3,3,1; 4,4,2; 2,2,2,4; N11: 4,4,2,1; 3,3,3,2; 3,4,3,1; N12: 3,3,3,3; 4,4,4; 4,2,3,3; N13: 5,5,3; 4,4,4,1; 4,3,4,2; N14: 4,4,3,3; 4,4,4,2; 3,3,3,5; N15: 4,4,4,3; 5,4,4,2; 5,5,4,1; N16: 4,4,4,4; 4,4,3,5; 5,5,4,2. On every session, all configurations were presented once, with subgroups randomly assigned to different quadrants and the squares randomly placed in one of the possible locations within each quadrant on every trial.

In the dual task conditions (again, performed separately with ungrouped and grouped stimuli), in addition to the numerosity estimation task, we tested the effect of cross-modal attentional load, using either auditory or visual distractors but also the effect of performing concurrent mathematical operations (thought to interfere with math-related groupitizing component) indicated by visual stimuli (Figure 1, panel E). All these stimuli were presented at the same time as the numerical arrays and lasted 500 ms. Participants performed three dual task conditions with auditory, visuo-spatial and arithmetic concurrent tasks (tested in separate sessions), responding to the concurrent task before providing the numerosity response (Figure 1D).

#### Auditory Task.

The auditory task (Aud-DT) consisted of an auditory time bisection and included a set of three 400 Hz auditory stimuli (each lasting 50 ms) presented through headphones. The first tone was presented when the array of squares was presented on screen (at 0 ms) and the third at the end of the stimulus presentation (at 500 ms; Figure 1E). The second tone was presented at a variable interval (70, 140, 215, 290, 360 430 ms) after the first sound. Participants had to indicate whether the middle sound was closer to the first or the third one using left (closer to the first tone) or right (closer to the third tone) arrow on the keyboard and then to verbally estimate the numerosity of the array.

#### Visuo-Spatial Task.

In the visuo-spatial task (Vsp-DT), a visual distractor was presented onscreen comprising four centrally positioned colored squares (each subtending 0.6° of visual angle, each distractor covered an area of 1.2° × 1.2°) which could have eight color arrangements (Figure 1E). Four color arrangements among the eight were chosen as targets. The stimuli targets were those that satisfied a specific conjunction of color and spatial arrangement: two green squares along the right diagonal, or two yellow squares along the left diagonal, while the squares along the opposite diagonal could be colored either in red or blue. Participants were first asked to indicate if the stimulus configuration was a target or a no-target using left (target) or right (no-target) arrow on the keyboard and then to verbally estimate the numerosity of the array.

#### Arithmetic Task.

In the arithmetic task (Arithm-DT) participants were asked to solve simple one-digit operations (for an example see Figure 1E). The stimuli (0.4° × 0.5° digits and 0.3° × 0.3° operand, Arial font) were placed at the center of the squares array. Participants verbally provided the result of the calculation and then estimated the numerosity of the array.

### Data Analysis

Data were separately analyzed for each participant. For the numerosity task we calculated the average perceived numerosity (accuracy) and standard deviation, separately for each numerosity, spatial arrangement and condition. Precision was measured by normalizing the standard deviation by the physical numerosity obtaining the Weber fraction (Wf), a dimensionless index of precision that allows comparison and averaging of performance across numerosities:

$$Wf=\frac{{SD}_{i}}{N}$$ (1)

where *SD*_*i*_ stands for the standard deviation of the responses to numerosity *i* and *N* stands for the analyzed numerosity.

Given that the distractors used in the visuo-spatial and arithmetic tasks were both visually presented, we quantified their specific contribution using principal component analysis (PCA). Wfs measured for the grouped stimuli in the Vsp-DT and Arithm-DT (numerosities between 5 and 8) were entered in a PCA and factors rotated with the oblique oblimin method. The number of components was determined as those exceeding an eigenvalue of 1.

Next, to compare the effect of attentional deprivation on numerical estimates across spatial arrangements, we averaged the Wfs across numerosities, to obtain a summary precision index for each condition. Specifically, for each participant and both grouped and ungrouped conditions, we calculated the attentional cost (*AC*) as the difference between Wfs in the dual and (DT) single task with ignored distractors (ST), normalized by their sum:

$$\text{Attentional}\;\text{cost}\;\left(AC\right)=\frac{{Wf}_{DT}-{Wf}_{ST}}{{Wf}_{ST}+{Wf}_{DT}}\times 100$$ (2)

where Wf_*ST*_ and Wf_*DT*_ are the average Weber fractions for the single with ignored distractors and dual task conditions.

For each participant we also measured the delta attentional cost (Δ*AC*) between grouped and ungrouped as:

$$\Delta AC={AC}_{G}-{AC}_{U}$$ (3)

Wfs, perceived numerosity and attentional cost were analyzed with Repeated Measures ANOVA and Bonferroni corrected post-hoc *t*-tests (*p*_bonf_). When the assumption of sphericity of the data was violated, we reported the resulting *p* value after Greenhouse-Geisser correction. Effect sizes associated with ANOVA were reported as *η*^2^, and those associated with post-hoc *t*-tests were reported as Cohen’s *d*. Frequentist statistics were supplemented with Bayesian statistics. Bayes Factors were calculated as the ratio of the likelihood of the alternative to the null hypothesis and reported as base ten logarithms (Log10 Bayes Factors, LBF). For RM-ANOVA we reported LBF_inclusion_ indicating how much the data are likely to occur from a model including that specific factor (or interaction), compared to models not including them. By convention, LBF > 0.5 is considered substantial evidence in favor of the alternative hypothesis and LBF < − 0.5 substantial evidence for the null hypothesis (no difference). Absolute values greater than 1 are considered strong evidence, and greater than 2 definitive evidence.

## RESULTS

### Effect of Grouping on Numerosity Estimation Precision (Single Task)

We first verified whether the results obtained in the single task (with and without ignored distractors presented on screen) replicated previous findings showing higher sensory precision (lower Wf) for grouped stimuli compared to ungrouped stimuli.

**Figure 2.**
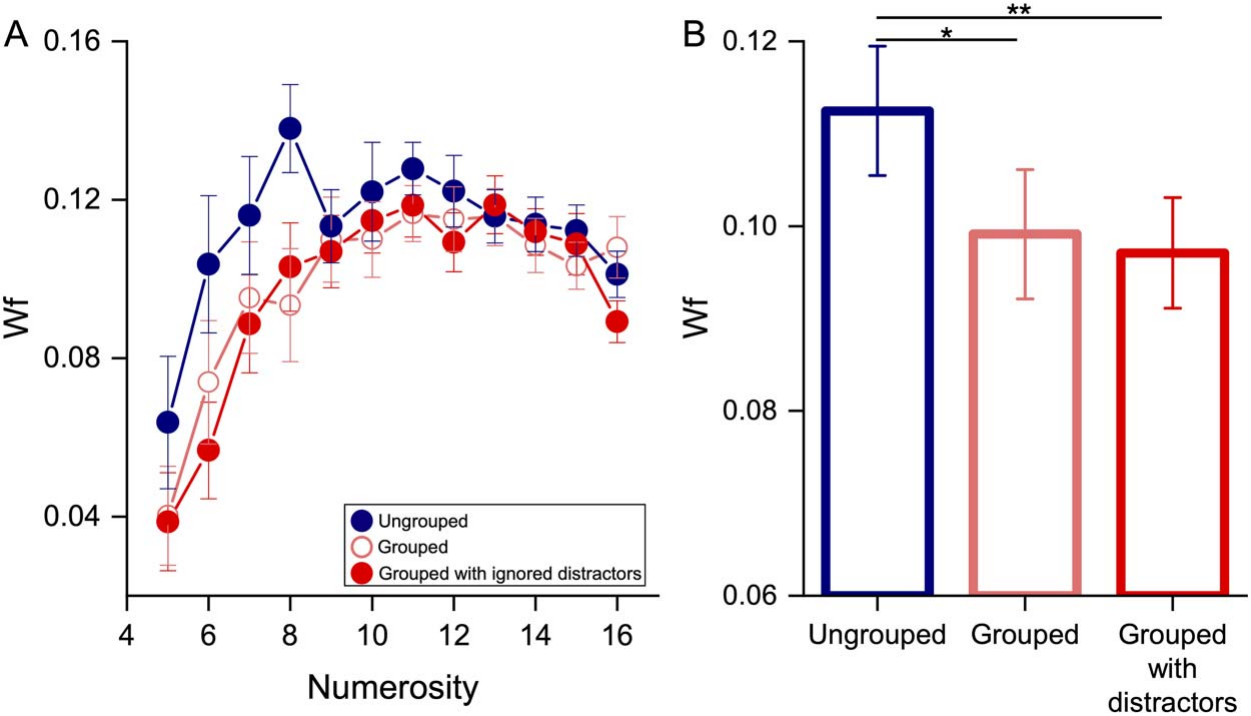
Groupitizing effect in the three single task conditions. (**A**) Average Weber fraction (Wf) as a function of numerosity for ungrouped (blue circles), grouped (pink circles) and grouped with distractors (red circles) conditions. (B) Weber fractions averaged across numerosities and participants. Error bars show ±1 *SEM*. *p_bonf_ = 0.016; **p_bonf_ < 0.01.

Figure 2A shows the average Wf as a function of numerosity for the three single tasks (ungrouped, grouped and grouped with ignored distractors). When only the stimuli for the numerosity task were presented onscreen, numerical estimates of grouped stimuli (pink empty circles; mean = 0.09, *SD* = 0.04) were more precise (lower Wfs) than those of ungrouped stimuli (blue circles; mean = 0.11, *SD* = 0.04), especially for lower numerosities, replicating previous results (Anobile et al., 2020). Displaying (ignored) distractors together with the white squares did not significantly change this result (mean = 0.09, *SD* = 0.03): even in this case, numerical estimates were more precise for grouped than ungrouped stimuli. A two-way Repeated measures ANOVA with numerosity (twelve levels: from 5 to 16) and single task condition (three levels: ungrouped, grouped and grouped with ignored distractors) as factors confirmed the significant main effect of single task condition (*p* = 0.005; see Table S1 for details) and that, regardless the presence of the (ignored) distractors, Wfs for grouped stimuli were lower than those for ungrouped stimuli (see pink and red bars vs. blue bar in Figure 2B, Table 1). The Wf decrease induced by groupitizing (regardless the presence of distractors) was clearest for the lower numerosities (up to eight items) and strongly decreased for higher numerosities (Figure 2A; interaction between numerosity and single task condition: *p* = 0.027; see Table S1 for details).

**Table 1.** Post-hoc *t*-tests between single task conditions.

**Single task condition comparison**	**df**	***t***	***p*_bonf_**	**Cohen’s *d***	**LBF**
Ungrouped vs. Grouped	27	2.91	0.016	0.25	1.74
Ungrouped vs. Grouped with ignored distractors	27	3.35	0.004	0.29	2.04
Grouped vs. Grouped with ignored distractors	27	0.44	>0.99	0.04	−0.66

Given that there was no difference between the precision measured in the two single tasks for grouped stimuli (no distractors vs. with distractors), in the following analysis we used data from the grouped with distractors condition as the reference performance for single task with grouped stimuli to match the visual stimulation across tasks. Moreover, we reduced the numerical range to those numerosities that showed the groupitizing advantage (up to N8; see Figure 2A).

### Effect of Attentional Load on Numerosity Estimation Precision (Dual Task)

We used a dual task paradigm to test the impact of depriving auditory or visual attention on sensory precision for ungrouped and grouped arrays. We also measured the interference effect of solving arithmetic operation concurrently to numerosity estimation of grouped and ungrouped stimuli.

**Figure 3.**
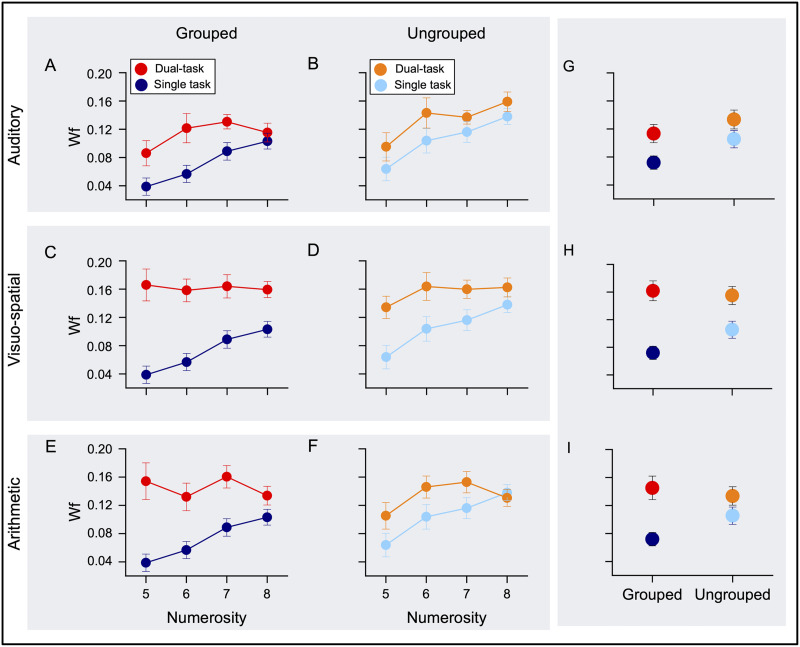
Attentional load effect on numerosity estimation precision. Average Weber fraction (Wf) as a function of numerosity for all experimental conditions. (**A**, **C**, **E**) Wfs for grouped stimuli (blue: single task, red: dual-task). (**B**, **D**, **F**) Wfs for ungrouped stimuli (light-blue: single task, orange: dual task). Wfs for single and auditory (**A**, **B**), visuo-spatial (**C**, **D**) and arithmetic (**E**, **F**) dual tasks. Average Wfs across numerosities and participants are reported for the single and the auditory (**G**), visuo-spatial (**H**) and arithmetic (**I**) dual tasks.

Figure 3 reported the average Wfs as a function of numerosity for grouped (Figures 3A, 3C, and 3E) and ungrouped stimuli (Figures 3B, 3D, and 3F) during the single and dual tasks. Depriving attention affected the precision of numerical estimates depending on the numerosity tested (more for lower than higher numerosities, Figure 3A–F) and the modality loaded (less when loading auditory attention and more when loading visual attention or solving arithmetic operations; Figure 3G–I; Table S2) for both grouped and ungrouped stimuli, although to a minor extent in the latter case.

To statistically test the impact of loading attention on the precision of numerical estimates, we performed two-way Repeated measures ANOVA with numerosity (four levels: from 5 to 8) and task (four levels: single task, Aud-DT, Vsp-DT, and Arithm-DT) as factors, for both grouped and ungrouped stimuli (see Table S3 and S6).

Performing any kind of dual task strongly increased Wfs for grouped stimuli compared to those measured in single task (see Table S3, main effect of task and post-hoc *t*-tests comparing ST vs. DTs in Table S4). In particular, interfering with visuo-spatial attention and arithmetic caused the highest Wf increase (2.3- and 2-fold increase from single task respectively, both post-hoc *t*-tests *p* < 0.0001; see also Table S2), while loading auditory attention had a smaller (1.6- fold increase, post-hoc *t*-test *p* = 0.006; see also Table S2), yet highly significant, effect on Wfs (see post-hoc *t*-tests comparing DTs in Table S4). The interaction between numerosity and task was significant (*p* = 0.002; see Table S3), suggesting that depriving attention modulated Wfs more for lower compared to higher numerosities.

Given that the distractors used in the visuo-spatial and arithmetic dual-task conditions were both presented visually they could have the same detrimental effects because generally tapping on visual resources, regardless of the underlying mechanism (visuo-spatial and arithmetic). To test this possibility, we performed a PCA on the Wfs measured in Vsp-DT and Arithm-DT for grouped stimuli (numerosities: 5–8). If the increases in Wfs for both dual tasks stem from the same, shared noise source (visual attention), the PCA should reveal a single component collapsing all the Wfs. Contrary to this prediction, the PCA revealed two principal components, accounting for 73% of the total variance (Table S5). More importantly, the first principal component (PC1) collapsed the Wfs measured in the Arithm-DT, while the second component (PC2) was mainly populated by Wfs measured in the Vsp-DT. This result is in line with the idea that the two different dual-tasks were tapping on (at least) partially independent noise sources.

Wfs measured in all dual tasks increased also for ungrouped stimuli compared to those measured in single task, although to a smaller extent (1.3-, 1.5-, 1.3- fold increase for Aud-DT, Vsp-DT and Arithm-DT respectively; all post-hoc *t*-tests *p* < 0.032; see Wfs in Table S2, main effect of task and post-hoc *t*-tests in Tables S6 and S7). Under dual tasks, Wfs increased equally for low and high numerosities, as suggested by the not significant interaction between numerosity and task (*p* = 0.07, LBF = 0.18; Table S5).

Overall, although the detrimental effect of attentional deprivation on sensory precision was observed for both grouped and ungrouped stimuli, it appeared more pronounced in the former case.

**Figure 4.**
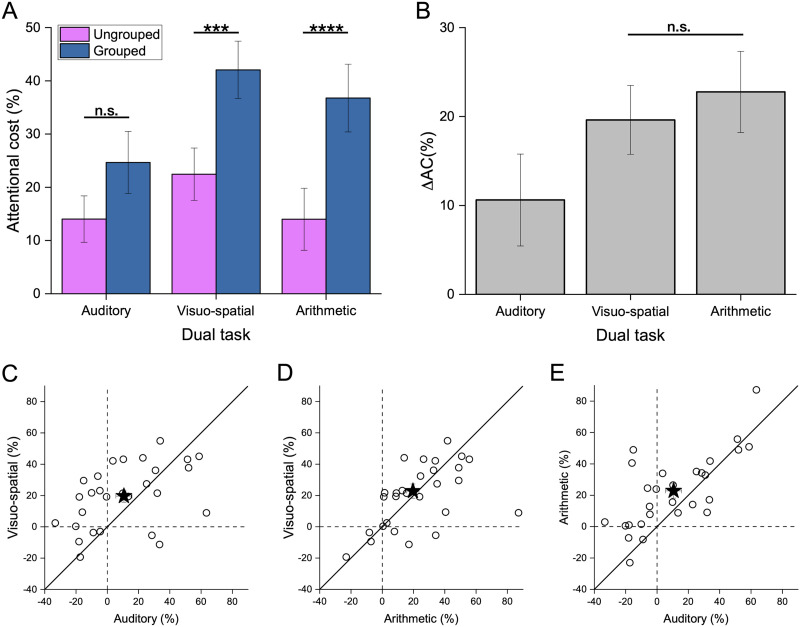
Attentional cost for each dual task condition and spatial arrangement. (**A**) Percentage of attentional cost for ungrouped (pink bars) and grouped (blue bars) spatial arrangements for auditory, visuo-spatial and arithmetic dual task conditions. (**B**) Delta attentional cost (Δ*AC*) for the auditory, visuo-spatial and arithmetic dual task conditions. (**C**) Δ*AC* for individual participants (circles) in the auditory dual task plotted as a function of Δ*AC* in the visuo-spatial dual task, (**D**) Δ*AC* for individual participants (circles) in the arithmetic dual task plotted as a function of Δ*AC* in the visuo-spatial dual task, and (**E**) Δ*AC* for individual participants (circles) in the auditory dual task plotted as a function of Δ*AC* in the arithmetic dual task. The black stars represent the average Δ*AC*. Error bars show ±1 *SEM*. ***p = 0.0009; ****p < 0.0001.

To quantify and compare the effect of attentional deprivation on numerical estimates across spatial arrangements we calculated the attentional cost (see Equation 2, Figure 4), which takes into account the better sensitivity for grouped stimuli when attentional resources were fully available (that is in single task). These values were entered in a two-way Repeated measures ANOVA with spatial arrangement (2 levels: grouped and ungrouped) and dual-task type (3 levels: Aud-DT, Vsp-DT and Arithm-DT) as factors. The significant main effect of spatial arrangement (*p* < 0.0001) confirmed that sensory precision was indeed higher when participants estimated grouped compared to ungrouped stimuli under dual tasks (see Table S8, Figure 4A). The interaction between spatial arrangement and dual-task type was also significant (*p* = 0.017; see Table S8) and post-hoc *t*-tests showed that loading attention by means of both visuo-spatial and arithmetic dual tasks had a stronger attentional cost on the precision of numerical estimates of grouped (Vsp-DT: 42 ± 28%; Arithm-DT: 37 ± 33%) compared to ungrouped (Vsp-DT: 22 ± 25%; Arithm-DT: 14 ± 30%) stimuli (see post-hoc *t*-tests in Table S9 comparing Grouped vs. Ungrouped Vsp-DT: *p* = 0.0009; Arithm-DT: *p* < 0.0001). On the contrary, loading auditory attention determined a smaller attentional cost for group stimuli, which did not differ from the one determined for ungrouped stimuli (25 ± 30% and 14 ± 23% for grouped and ungrouped respectively, post-hoc *t*-test Grouped vs. Ungrouped Aud-DT: *p* = 0.328; see Table S9). Figure 4B shows more clearly these results: we subtracted the attentional cost of ungrouped from that of grouped condition (difference between blue and pink bars in Figure 4A), obtaining a delta index of the attentional cost (Δ*AC*). Positive Δ*AC* values indicate that the attentional cost for grouped stimuli was larger than that for ungrouped stimuli. This was the case for all conditions, although the Δ*AC* significantly differed from zero (alpha level = 0.05/3 = 0.017) only when loading visuo-spatial and arithmetic attention (both *t*-tests against 0: *p* < 0.001), and not when loading auditory attention (in line with the differences between the pink and blue bars in Figure 4A, see Table 2, *t*-tests against 0: *p* = 0.046). Importantly, the Δ*AC* for the arithmetic and visuo-spatial conditions did not differ (see Figure 4C–E for single subjects’ scatter and Table S10; *t*-test Δ*AC* Vsp-DT vs. Δ*AC* Arithm-DT: *p* = 0.45), suggesting that these components are equally crucial in determining the groupitizing advantage.

**Table 2.** One sample *t*-tests of Δ*AC* (against zero).

**Condition**	**Mean**	**std**	**df**	***t***	***p***	**Cohen’s *d***	**LBF**
Auditory	10.61	5.17	27	2.09	0.046	0.39	0.12
Visuo-spatial	19.6	3.9	27	5.16	<0.001	0.97	>3.00
Arithmetic	22.8	4.6	27	5.07	<0.001	0.96	3.00

Taken together these results suggested that depriving visuo-spatial attention and performing concurrent arithmetic computations interfered more with numerosity estimation of grouped than ungrouped stimuli, while loading auditory attention had an overall smaller and similar impact on numerosity estimation irrespective of the spatial arrangements.

### Effect of Grouping and Attentional Load on Perceived Numerosity (Accuracy)

**Figure 5.**
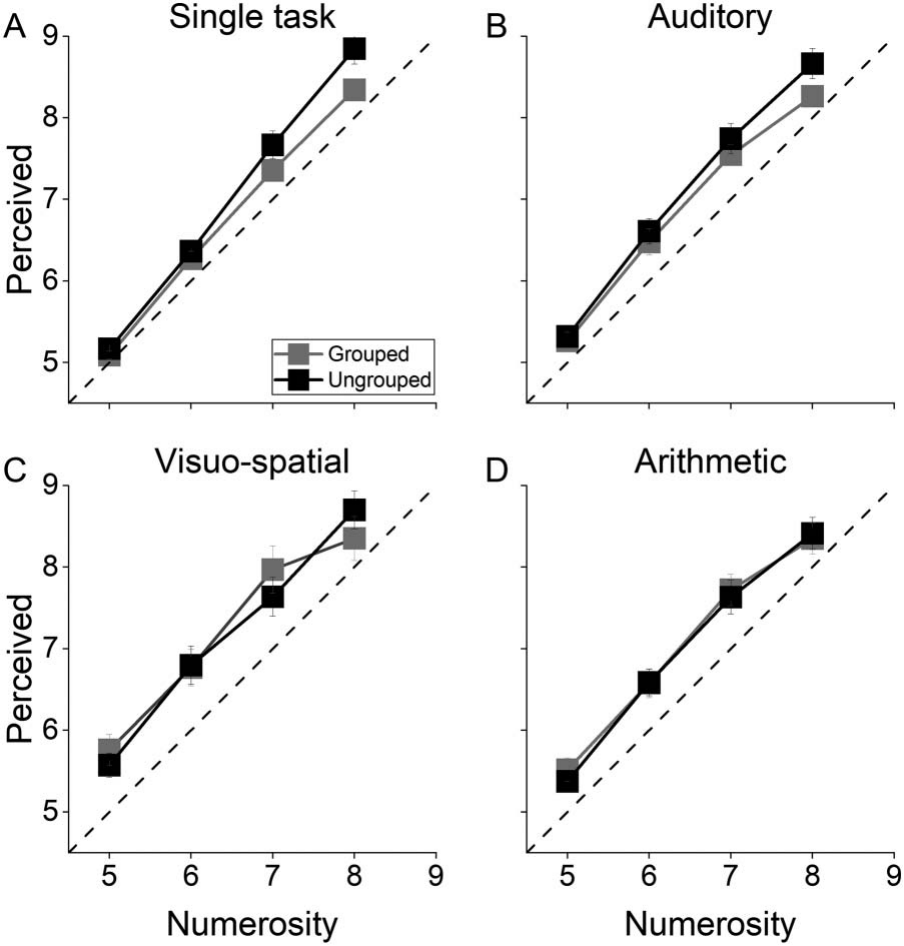
Average perceived numerosity for grouped (gray squares) and ungrouped (black squares) stimuli for numerosities between 5 and 8. (**A**) Average perceived numerosity as a function of physical numerosity for the single task, auditory (**B**), visuo-spatial (**C**), and arithmetic (**D**) dual task conditions. Error bars show ±1 *SEM*.

We also evaluated the effect of grouping and task on perceived numerosity (Table 3) to measure if any of the effects reported for the precision of numerosity estimates also generalized to the estimate’s accuracy. Figure 5 shows the average responses as a function of the physical numerosity separately for single task (Figure 5A), and the three dual task conditions (auditory: Figure 5B; visuo-spatial: Figure 5C, and arithmetic: Figure 5D).

**Table 3.** Descriptive statistics of average perceived numerosity.

**Attentional load**	**Grouping**	**Mean**	**std**
Single task	Ungrouped	6.97	0.58
	Grouped	6.76	0.39
Auditory dual task	Ungrouped	7.04	0.66
	Grouped	6.88	0.56
Visuo-spatial dual task	Ungrouped	7.18	1.10
	Grouped	7.19	1.22
Arithmetic dual task	Ungrouped	6.94	0.74
	Grouped	7.01	0.82

To statistically test differences across conditions, we performed a three-way Repeated measures ANOVA with numerosity (four levels: from 5 to 8), spatial arrangement (two levels: grouped and ungrouped) and task (four levels: single task, Aud-DT, Vsp-DT, and Arithm-DT) as factors. There was no significant main effect of spatial arrangement (*p* = 0.17) nor of task (*p* = 0.09; see Table S11), suggesting that none of these factors impacted on perceived numerosity.

### Concurrent Task Performance

To verify that the concurrent tasks were accurately performed during the dual task conditions, we measured the average performance for each participant. Indeed, the effects of different dual tasks (auditory, visual or arithmetic) might be accounted for in terms of an imbalance in attentional deployment between the primary and the distractor tasks in the different conditions. The accuracies for the concurrent task averaged across participants and grouping conditions for numerosities from 5 to 8 were: 82% ± 11% for auditory, 94% ± 6% for visuo-spatial and 96% ± 5% for arithmetic tasks. The fact that the visuo-spatial and arithmetic tasks were those generating the strongest interference with the precision of numerosity estimation of grouped stimuli is striking given that these tasks seem to be the easiest (highest accuracy) compared to the less-interfering auditory task.

## DISCUSSION

Groupitizing allows individuals to estimate the number of objects more precisely when these are clustered rather than randomly scattered in space (Anobile et al., 2020, 2021; Ciccione & Dehaene, 2020; Guillaume et al., 2023; Maldonado Moscoso et al., 2020; Starkey & McCandliss, 2014). In the current study we found that groupitizing occurs despite increased sensory load, that is, despite the presence of irrelevant visual and auditory distractors onscreen (single task with distractors). Previous evidence suggested that groupitizing might leverage on two mechanisms: the recruitment of the subitizing system and the possibility to perform arithmetic operations on the subitized groups. In the present work, we tested this hypothesis by interfering with both these components through dual-task paradigms: on the one hand we loaded auditory or visuo-spatial attention known to be crucial for subitizing (Anobile et al., 2012); on the other hand, we interfered with calculation.

We found that the attentional cost of performing any concurrent task was overall higher for grouped compared to ungrouped stimuli, in line with the possible recruitment of subitizing-related attentional resources during groupitizing. Previous studies using dual task (Burr et al., 2010; Pomè, Anobile, Cicchini, & Burr, 2019; Vetter et al., 2008), attentional blink (Burr et al., 2010; Egeth et al., 2008; Olivers & Watson, 2008; Xu & Liu, 2008), inattentional blindness (Railo et al., 2008), adaptation (Burr et al., 2011) and eye-tracking (Castaldi et al., 2020) paradigms showed that subitizing requires attentional resources to operate (see Chen et al., 2022 for a recent review and meta-analysis). On the contrary, the ANS system seems to be less susceptible, although not completely immune to attentional deprivation (Castaldi, Piazza, et al., 2021; Pomè, Anobile, Cicchini, Scabia, et al., 2019). Given that the numerosities tested in the current experiment were all higher than those typically processed by the subitizing system, the stronger attentional modulation observed for grouped than ungrouped arrays strongly suggests that the concurrent tasks interfered with an attention-dependent mechanisms in the former case, likely the subitizing system. Burr et al. (2010) suggested that the same mechanism for numerosity perception might operate over the whole numerical range, but that an attention-based system (subitizing) can be called on to process small numbers with higher precision. EEG studies also supported the possibility that the ANS might operate over the entire numerical range, including the subitizing range, and that this can be best appreciated when attention is diverted. Indeed, while under full attention conditions, viewing small numerosities only modulated an early negative component of evoked potentials (the N1 component also modulated by groupitizing; Caponi et al., 2023; Hyde & Spelke, 2009, 2012; Mazza et al., 2013), viewing them under high attentional load, additionally modulated the amplitude of a later component, the P2p, which instead typically scales with the numerosity of larger arrays (Hyde & Wood, 2011). Overall, these results suggest that an estimation mechanism might operate across all numerical ranges, but the low numerosities in the subitizing range benefit from an additional attentional-demanding mechanism. The current results expand this conclusion, by showing that this mechanism can be activated also when processing higher numerosities, higher than the typical 4-items limit, provided that grouping cues are available, giving rise to the groupitizing phenomenon. The current study, thus, provides further evidence of the strong and multiple interplay between the subitizing and ANS systems.

Interestingly, we found that while performing any kind of dual task strongly enhanced sensory precision for grouped stimuli, only interfering with visuo-spatial attention and arithmetic abilities, but not with auditory attention, impacted the precision of numerical estimates of grouped stimuli more than what it did for those measured for ungrouped stimuli. This result cannot be explained by a difficulty difference in the concurrent task, being the auditory task even more difficult than the visuo-spatial task (∼83% vs. ∼94% correct response for the auditory and visuo-spatial task respectively). A more likely explanation is that numerosity perception might rely more on visuo-spatial than auditory attention. Pomè, Anobile, Cicchini, Scabia, et al. (2019) reported that sensory precision in the subitizing range was more affected by visuo-spatial than auditory attentional deprivation and the same trend was observed, although to a minor extent, also for larger numerosities processed by the ANS. When processing grouped arrays of large numerosities (>4 items), as in the case of the current experiment, both the subitizing and the ANS are likely activated and their common stronger reliance on visuo-spatial than auditory attentional resources might sum up, resulting in higher attentional cost during the visuo-spatial than the auditory task.

Overall, these results suggest that groupitizing, as subitizing, relies on cross-modal attention, although it might rely more on its visuo-spatial than auditory component.

In the present study, we also found that an easy arithmetic calculation task (average performance: ∼97%) was able to strongly impair the precision of numerical estimates of grouped stimuli, more than what it did for ungrouped stimuli. Interestingly, covariance analyses suggest that the worsening in sensory precision observed when estimating grouped stimuli under the arithmetic dual-task did not tap on the same resources used by the visual-spatial dual task. As both dual tasks were visually presented, this latter result suggests that their detrimental effects were specific and not derived from a general deprivation of visual attentional resources. This interference effect supports the idea that during groupitizing the subitized subgroups are summed/multiply by each other producing precise estimate of the total array numerosity, while numerosity perception of ungrouped stimuli occurs at a glance and without performing precise arithmetic calculation or counting. By asking participants to solve unrelated arithmetical operations while estimating the numerosity of grouped stimuli, we interfered with this process and strongly deteriorated the precision of numerical estimates. This is in line with previous evidence showing that the groupitizing advantage can be observed only after the acquisition of arithmetic competencies (Starkey & McCandliss, 2014) and that estimating grouped numerosities activate cortical areas often recruited during arithmetic calculations in adults (Maldonado Moscoso, Greenlee, et al., 2022). Despite this evidence for the role of math on groupitizing, the few available clinical studies on dyscalculia provide not clear cut results. Anobile et al. (2022) with children and later Gilstron et al. (2024) with adults both found a similar groupitizing advantage in dyscalculia, compared to controls clearly calling for further investigations (however see also Durgin et al., 2022 for possible methodological aspects).

As for perceived numerosity (accuracy), we observed that different spatial configurations of the stimuli did not lead to consistent bias in numerical estimates towards an over or underestimation. This seems to be in contrast with previous studies that have examined the impact of Gestalt cues, such as proximity, similarity, symmetry, and connectedness on numerosity perception (Adriano & Ciccione, 2024; Adriano et al., 2021, 2022; Anobile et al., 2017; Apthorp & Bell, 2015; Bertamini et al., 2016; Castaldi, Pomè, et al., 2021; Fornaciai et al., 2016; Franconeri et al., 2009; He et al., 2009; Kirjakovski & Matsumoto, 2016; Maldonado Moscoso et al., 2023; Maldonado Moscoso, Anobile, et al., 2022; Poom et al., 2019; Yu et al., 2019). Methodological differences are likely to explain this discrepancy. For example, studies reporting numerosity biases induced by Gestalt cues generally asked participants to compare/discriminate stimuli shown in the periphery, while groupitizing is usually measured using estimation of central stimuli. This may prompt participants to provide more accurate numerical estimates. To our knowledge, only one study reported numerosity underestimation using an estimation task when items were grouped (Adriano et al., 2021). However, in that study, Adriano et al. (2021) used Gestalt cues creating a sort of connectedness illusion (e.g., Pac-man-like stimuli), which is one of the strongest underestimation effects (up to 30%), likely stronger than that elicited by proximity which may be (if anything) in act here. Moreover, the numerosities tested are usually higher than those used to elicit groupitizing. Future studies should investigate the impact of these methodological differences on numerical biases induced by Gestalt cues.

In conclusion, the current study suggests that when individuals estimate numerosity, they activate different strategies depending on the spatial configuration of the items in the array: when items are grouped in space, the ANS system might benefit from the recruitment of visuo-spatial attentional resources and arithmetic calculation to enhance the precision of numerical estimates.

## FUNDING INFORMATION

This research was funded by the European Union—Next GenerationEU (PRIN 2022, Project ‘RIGHTSTRESS—Tuning arousal for optimal perception’, grant No. 2022CCPJ3J, CUP: B53D23014530001.

## AUTHOR CONTRIBUTIONS

Conceptualization: P.A.M.M.; G.A.; R.A.; E.C. Formal analysis: P.A.M.M. Investigation: P.A.M.M.; G.M. Methodology: P.A.M.M.; E.C.; G.A. Writing – original draft: P.A.M.M. Writing – review & editing: G.A.; R.A.; E.C. All authors approved the final version of the manuscript for submission.

## DATA AVAILABILITY STATEMENT

Data for the main findings are available at: https://doi.org/10.5281/zenodo.14599221.

## Supplementary Material


