# Supplementary material for: “Groupitizing”: A Visuo-Spatial and Arithmetic Phenomenon
Source: Open Mind (Camb). 2025 Jan 20;9:121–37. doi: 10.1162/opmi_a_00181 (PMC11774540; doi:10.1162/opmi_a_00181)
Supplement: Supplementary file 1 [file opmi-09-121-s001.docx]

**Table S1:** RM ANOVA (numerosity x single task conditions) on single task Weber fractions, summary table.

| **Parameter** | **df** | **F** | **p** | **η^2^** | **LBF** |
| --- | --- | --- | --- | --- | --- |
| Numerosity | 11, 297 | 12.864 | <0.0001 | 0.180 | >3.00 |
| Single tasks | 2, 54 | 6.627 | 0.005 | 0.021 | 1.13 |
| Numerosity*Single tasks | 22, 594 | 2.034 | 0.027 | 0.023 | 0.91 |

**Table S2:** Descriptive statistics of Weber fractions.

| **Condition** | **mean** | **std** |
| --- | --- | --- |
| **Single task (N:5-8)** |  |  |
| Grouped with ignored distractor | 0.07 | 0.05 |
| Ungrouped | 0.10 | 0.06 |
| **Dual task grouped** |  |  |
| Auditory | 0.11 | 0.06 |
| Visuo-spatial | 0.16 | 0.07 |
| Arithmetic | 0.14 | 0.09 |
| **Dual task ungrouped** |  |  |
| Auditory | 0.13 | 0.07 |
| Visuo-spatial | 0.15 | 0.07 |
| Arithmetic | 0.13 | 0.07 |

**Table S3:** RM ANOVA (numerosity x task) on Weber fractions for grouped stimuli, summary table.

| **Parameter** | **df** | **F** | **p** | **η^2^** | **LBF** |
| --- | --- | --- | --- | --- | --- |
| Numerosity | 3, 81 | 3.526 | 0.037 | 0.020 | 1.98 |
| Task | 3, 81 | 23.659 | <0.0001 | 0.249 | >3.00 |
| Numerosity* Task | 9, 243 | 3.748 | 0.002 | 0.036 | 2.44 |

**Table S4:** Post-hoc t-tests of main effect of task on Weber fractions for grouped stimuli, summary table.

| **Task** | **df** | **t** | **p_bonf_** | **Cohen’s d** | **LBF** |
| --- | --- | --- | --- | --- | --- |
| **Single task** |  |  |  |  |  |
| Auditory dual task | 27 | –3.43 | 0.006 | –0.47 | 2.36 |
| Visuo-spatial dual task | 27 | –7.78 | <0.0001 | –1.08 | >3.00 |
| Arithmetic dual task | 27 | –6.33 | <0.0001 | –0.88 | >3.00 |
| **Auditory dual task** |  |  |  |  |  |
| Visuo-spatial dual task | 27 | –4.35 | 0.0002 | –0.60 | >3.00 |
| Arithmetic dual task | 27 | –2.90 | 0.028 | –0.40 | 0.57 |
| **Visuo-spatial** **dual task** |  |  |  |  |  |
| Arithmetic dual task | 27 | 1.45 | 0.91 | 0.20 | –0.27 |

**Table S5:** Component loadings of the PCA performed on Wfs of grouped stimuli in the visuo-spatial and Arithmetic dual-task (numerosities: 5-8). Loadings above 0.4 displayed.

| Variables | PC1 | PC2 |
| --- | --- | --- |
| 7 Arithmetic | **0.985** | –0.105 |
| 8 Arithmetic | **0.914** | –0.018 |
| 6 Arithmetic | **0.871** | 0.07 |
| 5 Arithmetic | **0.650** | 0.331 |
| 7 Visuo-spatial | 0.470 | **0.477** |
| 5 Visuo-spatial | 0.318 | **0.686** |
| 6 Visuo-spatial | –0.107 | **0.942** |
| 8 Visuo-spatial | 0.011 | **0.842** |

**Table S6:** RM ANOVA (numerosity x task) on Weber fractions for ungrouped stimuli, summary table.

| **Parameter** | **df** | **F** | **p** | **η^2^** | **LBF** |
| --- | --- | --- | --- | --- | --- |
| Numerosity | 3, 81 | 11.004 | <0.0001 | 0.094 | >3.00 |
| Task | 3, 81 | 8.581 | 0.0002 | 0.081 | 2.55 |
| Numerosity* Task | 9, 243 | 1.874 | 0.074 | 0.022 | 0.18 |

**Table S7:** Post-hoc t-tests of main effect of task on Weber fractions for ungrouped stimuli, summary table.

| **Task** | **df** | **t** | **p_bonf_** | **Cohen’s d** | **LBF** |
| --- | --- | --- | --- | --- | --- |
| **Single task** |  |  |  |  |  |
| Auditory dual task | 27 | –2.86 | 0.032 | –0.34 | 1.18 |
| Visuo-spatial dual task | 27 | –5.05 | <0.0001 | –0.60 | 2.83 |
| Arithmetic dual task | 27 | –2.87 | 0.031 | –0.34 | 0.74 |
| **Auditory dual task** |  |  |  |  |  |
| Visuo-spatial dual task | 27 | –2.19 | 0.19 | –0.26 | –0.01 |
| Arithmetic dual task | 27 | –0.01 | >0.99 | <–0.01 | –0.70 |
| **Visuo-spatial** **dual task** |  |  |  |  |  |
| Arithmetic dual task | 27 | 2.18 | 0.19 | 0.26 | –0.03 |

**Table S8:** RM ANOVA (spatial arrangement x dual task type) on attentional cost, summary table.

| **Parameter** | **df** | **F** | **p** | **η^2^** | **LBF** |
| --- | --- | --- | --- | --- | --- |
| Spatial arrangement | 1, 27 | 22.091 | <0.0001 | 0.197 | 2.91 |
| Dual task | 2, 54 | 5.099 | 0.009 | 0.070 | 1.08 |
| Spatial arrangement *Dual task | 2, 54 | 4.428 | 0.017 | 0.017 | 0.92 |

**Table S9:** Post-hoc t-tests of interaction between spatial arrangement and dual task type on attentional cost, summary table.

| **Dual-task** | **df** | **t** | **p_bonf_** | **Cohen’s d** | **LBF** |
| --- | --- | --- | --- | --- | --- |
| **Grouped auditory** |  |  |  |  |  |
| Ungrouped auditory | 27 | 2.37 | 0.328 | 0.37 | 0.12 |
| Grouped visuo-spatial | 27 | –3.81 | 0.004 | –0.61 | 2.37 |
| Grouped arithmetic | 27 | –2.65 | 0.144 | –0.42 | 0.31 |
| **Grouped visuo-spatial** |  |  |  |  |  |
| Ungrouped visuo-spatial | 27 | 4.37 | 0.0009 | 0.69 | >3.0 |
| Grouped arithmetic | 27 | 1.16 | >0.99 | 0.19 | –0.45 |
| **Grouped arithmetic** |  |  |  |  |  |
| Ungrouped arithmetic | 27 | 5.08 | <0.0001 | 0.80 | 2.96 |
| **Ungrouped auditory** |  |  |  |  |  |
| Ungrouped visuo-spatial | 27 | –1.84 | >0.99 | –0.295 | 0.17 |
| Ungrouped arithmetic | 27 | 0.01 | >0.99 | 0.002 | –0.70 |
| **Ungrouped visuo-spatial** |  |  |  |  |  |
| Ungrouped arithmetic | 27 | 1.85 | >0.99 | 0.30 | –0.17 |

**Table S10:** Paired samples t-test on $\Delta AC$, summary table.

| **Condition** | **df** | **t** | **p_bonf_** | **Cohen’s d** | **LBF** |
| --- | --- | --- | --- | --- | --- |
| Auditory vs Visuo-spatial | 27 | –1.87 | 0.072 | –0.35 | –0.04 |
| Auditory vs Arithmetic | 27 | –3.26 | 0.003 | –0.62 | 1.11 |
| Visuo-spatial vs Arithmetic | 27 | –0.77 | 0.45 | –0.44 | –0.58 |

**Table S11:** RM ANOVA (numerosity x spatial arrangement x task) on perceived numerosity, summary table.

| **Parameter** | **df** | **F** | **p** | **η^2^** | **LBF** |
| --- | --- | --- | --- | --- | --- |
| Numerosity | 3, 81 | 890.915 | <0.0001 | 0.795 | >3.00 |
| Spatial arrangement | 1, 27 | 1.975 | 0.171 | 0.0007 | 2.42 |
| Task | 3, 81 | 2.654 | 0.096 | 0.008 | >3.00 |
| Numerosity*Spatial arrangement | 3, 81 | 9.187 | 0.0001 | 0.004 | 2.91 |
| Numerosity*Task | 9, 243 | 6.246 | <0.0001 | 0.005 | >3.00 |
| Spatial arrangement * Task | 3, 81 | 3.076 | 0.052 | 0.002 | 0.63 |
| Numerosity*Spatial arrangement *Task | 9, 243 | 1.810 | 0.095 | 0.001 | 0.60 |
